# Coronavirus disease 2019 outcomes in heart transplant recipients: a single-center case series

**DOI:** 10.1186/s13256-021-03028-5

**Published:** 2021-09-13

**Authors:** Farah Naghashzadeh, Shadi Shafaghi, Babak Sharif-Kashani, Payam Tabarsi, Leila Saliminejad, Sima Noorali

**Affiliations:** 1 Lung Transplantation Research Center, National Research Institute of Tuberculosis and Lung Diseases (NRITLD), Shahid Beheshti University of Medical Sciences, Tehran, Iran; 2 Tobacco Prevention and Control Research Center, National Research Institute of Tuberculosis and Lung Diseases (NRITLD), Shahid Beheshti University of Medical Sciences, Tehran, Iran; 3 Infectious Disease Specialist, Fellowship of Infection in Immunocompromised Host, Clinical Tuberculosis and Epidemiology Research Center, National Research Institute of Tuberculosis and Lung Diseases (NRITLD), Shahid Beheshti University of Medical Sciences, Tehran, Iran; 4 Bachelor of Science in Nursing (BSN), Lung Transplantation Research Center, National Research Institute of Tuberculosis and Lung Diseases (NRITLD), Shahid Beheshti University of Medical Sciences, Tehran, Iran

**Keywords:** COVID-19, Heart transplantation, Solid-organ transplantation, Immunosuppression

## Abstract

**Background:**

With the rapidly expanding pandemic of severe acute respiratory syndrome coronavirus-2, a chronic immunosuppressed state in solid organ transplant recipients is a concern. We reported coronavirus disease 2019 in heart transplant recipients and described the patients’ course from diagnosis to either hospital admission or improvement in symptoms.

**Case presentation:**

This study retrospectively identified 13 white (Iranian) heart transplant patients with coronavirus disease 2019 between December 2019 and October 2020. The mean age of patients was 43.7 years (19–65 years); seven (70%) were men. Laboratory and treatment data were collected for those admitted or managed as outpatients. Outcomes were also recorded for all patients. This report demonstrates a range of symptoms, clinical severity, and disease course in heart transplant recipients with coronavirus disease 2019, including ten hospitalized patients and three patients, managed entirely in the outpatient setting. One patient passed away, and none of them experienced an episode of clinically overt rejection.

**Conclusions:**

We would like to emphasize the importance of being alert in these patients to consider testing in a broad range of clinical presentations and gathering more data for better management.

## Introduction

The recently novel coronavirus, severe acute respiratory syndrome coronavirus-2 (SARS-CoV-2), emerged in China in December 2019 and caused a highly contagious disease called coronavirus disease 2019 (COVID-19) [1]. In less than 3 months, it spread throughout the globe and was declared a global pandemic by the World Health Organization on 11 March 2020 [2]. To date, COVID-19 has affected over 9 million people worldwide. The clinical manifestations of COVID-19 range from minimally symptomatic individuals to severe courses with the requirement of invasive mechanical ventilation and multiorgan failure [3, 4].

Risk factors for morbidity and mortality include older age, cardiovascular disease, diabetes, chronic respiratory disease, hypertension, obesity, and cancer. A particular concern also exists regarding the vulnerability of pharmacologically immunosuppressed patients before or during this pandemic [3, 5]. Thus, solid organ transplant (SOT) recipients, including heart transplant (HTx) recipients, are considered to be vulnerable to COVID-19 infection because of immunosuppression and the presence of comorbidities. Epidemiologic and clinical characteristics of HTx recipients during the SARS-CoV-2 pandemic are largely unknown [6].

The following report describes 13 cases of HTx recipients with laboratory‐confirmed SARS‐CoV‐2 and reviews the clinical presentations, treatment strategies, and short‐term outcomes.

## Case presentation

### Patient population

In this single-center case series of 135 recipients of HTx, 25 confirmed COVID-19 recipients from December 2019 to October 2020 were presented, but information of only 13 patients was available. Demographic data including age, sex, and medical history were collected for each patient. Clinical presentations and laboratory data, therapeutic management, and outcomes were collected and analyzed for those admitted to our hospital or managed as outpatients. Nasopharyngeal swab RT-PCR testing was done in all of the patients with first mild symptoms and also in patients who were in touch with definite COVID-19 patients.

### Statistical analyses

All quantitative variables are expressed as means.

## Results

### Demographic data

In this case series, we presented 13 patients with laboratory‐confirmed SARS‐CoV‐2. The mean age of patients was 43.7 years (19–65 years); seven (70%) were men. Previous history of diabetes mellitus was most frequently reported (three patients, 37.5%). Three patients (25%) had hypertension, and two patients had chronic kidney disease (Table 1).

**Table 1:** Patient characteristics, symptoms, treatment, and outcomes

Demographics	No.
Total, no.	13
Age, mean (years)	44.3
Male	9
Time, post-transplant, mean (years)	6.4
Hypertension	3
Diabetes mellitus	3
Chronic kidney disease^a^	2
Maintenance immunosuppression	
Total, no.	13
Tacrolimus	10
Cyclosporine	3
Mycophenolate mofetil	6
Mycophenolate sodium	7
Methylprednisolone	5
No. of immunosuppressive medications	
3	13
Symptoms	
Total, no.	11
Asymptomatic	2
Fever	6
Myalgia or malaise	5
Shortness of breath or cough	7
Gastrointestinal symptoms	3
Treatment	
Total inpatients and outpatients, no.	13
Remdesivir	9
Tocilizumab	1
Interferon beta	1
Intravenous immunoglobulin	2
Dexamethasone	7
Reduction in immunosuppression medications	8
Mycophenolate mofetil held	9
Supplemental oxygen	6
Outcome	
Total, no.	13
Overall survival	12
Overall mortality	1
Hospitalized	10
In-hospital mortality	1
ICU admission	2
Discharged	12
Managed as outpatient	3
Clinically overt rejection	0

^a^Defined by a glomerular filtration rate less than 30

### Clinical presentation and laboratory findings

The mean time between the transplantation and COVID-19 diagnosis was 6.4 years (1–12 years). Clinical presentations included fever (6, 50%), dyspnea or cough (7, 53.8%), myalgia or malaise (5, 38.4%), and gastrointestinal symptoms (3, 23%); and two (15.3%) patients were asymptomatic. Ten patients (76.9%) were admitted, three (23%) were managed as outpatients, and two (15.3%) of the inpatients required admission to the intensive care unit (ICU) (Table 1).

Notably, mean white blood cell count was about 4500/μL (1500–8000/μL), with a mean absolute lymphocyte count of 1000/μL (300–3200/μL). High-sensitivity C-reactive protein and erythrocyte sedimentation rate were greater than normal in all admitted patients. One patient had severe creatinine elevation termed acute on chronic kidney failure. All patients had a normal echocardiogram during hospitalization.

### Treatment and outcome

The therapeutic management involved an immunosuppressive regimen and supportive care in 13 patients. Supplemental oxygen was required in six of the hospitalized patients. Routine immunosuppressive regimens in HTx recipients included calcineurin inhibitors (cyclosporine, tacrolimus), mycophenolate, and corticosteroid. Three patients received cyclosporine, tacrolimus was administered in ten, mycophenolate mofetil in six, mycophenolate sodium in seven, and methylprednisolone in five cases. During infection with COVID-19, mycophenolate was discontinued in nine admitted patients for at least 1 week, and the dosage of calcineurin inhibitors was reduced to a lower limit of suitable blood level for transplant age in eight cases. All of the patients received azithromycin, nine admitted patients with pulmonary involvement received remdesivir 200 mg stat on the first day, and then 100 mg daily for four consecutive days. Tocilizumab was administered in one patient and interferon beta in another one. Two patients underwent intravenous immunoglobulin therapy, seven patients with mild pulmonary involvement received intravenous dexamethasone instead of oral prednisolone, and five patients with moderate-to-severe pulmonary involvement received pulse therapy with methylprednisolone.

Twelve patients survived, but one of the ICU admitted patients underwent hemodialysis and finally died from respiratory and kidney failure. None of the survived patients experienced an episode of clinically overt rejection (Table 1).

## Discussion

The COVID‐19 pandemic has presented the medical community with unique challenges and severely impacts large parts of the world. Further development of this disease cannot be predicted, and especially knowledge regarding outcomes of at‐risk patients such as transplant recipients is still evolving [7]. The immunosuppressants used to prevent and treat rejection in transplant recipients leave patients susceptible to infectious complications, of which pulmonary infections are the leading cause of morbidity and mortality [8].

The first case of COVID-19 in HTx was a 51-year-old man with a severe presentation from China [9]. Available reports of COVID‐19 in SOT recipients from China, Italy, France, Germany, the USA, and Spain highlight variable presentations and outcomes, despite immunosuppression [4, 10–21]. There are no published reports of COVID‐19 in the heart transplant recipient population in Iran. This study reviews clinical parameters and outcomes of 13 cases of HTx recipients with laboratory‐confirmed SARS‐CoV‐2 followed by our transplant center in Tehran, Iran. These cases presented with a range of severity of clinical presentations and course of hospitalization, including ten patients requiring admission and three managed as outpatients. Despite risk factors for severe disease, including older age, immunosuppression, and other comorbidities, most of the patients had mild symptoms, but one (7.6%) of them died from respiratory and kidney failure. In a study conducted in Spain with 18 SOT [kidney (44.4%), liver (33.3%), and heart (22.2%)] recipients diagnosed with COVID-19, the case fatality rate was 27.8% [22]. Based on another cohort from the USA, with 90 patients, the mortality rate was 18% [23].

The general approach followed in the published reports was to decrease or hold the immunosuppressant [7]. In this series, we held mycophenolate mofetil in six patients, and this was due to concern for the worsening of COVID‐19 disease. Despite all require maintenance immunosuppression that predisposes recipients to greater infectious risk, immunosuppression has also been theorized to be protective against cytokine storm [24]. It should be noted as a limitation that this case series presents a small number of patients, which limits the ability to evaluate the efficacy of the treatment strategy. It will certainly be beneficial to collaborate with other high-volume centers to gather more data on the disease and gain better insight into the clinical course and optimal management of this disease in these immunocompromised patients.

## Conclusion

We presented our experience of HTx recipients who acquired the SARS‐CoV‐2 virus at a tertiary care transplant center. This study reflected the diagnosis and management of COVID‐19 in immunocompromised patients. We would like to emphasize the importance of being alert in these patients to consider testing in a broad range of clinical presentations—even in asymptomatic patients that have been in contact with definite cases—and gathering more data for better management.

## Data Availability

The data are available in Table 1.

## Abbreviations

COVID‐19: Coronavirus disease 2019
HTx: Heart transplant recipients
SARS‐CoV‐2: Severe acute respiratory syndrome coronavirus-2
SOT: Solid organ transplantation

## References

[1] Ahmadi ZH, Mousavizadeh M, Nikpajouh A, Bahsir M, Hosseini S (2020). COVID-19: a perspective from Iran. J Card Surg.

[2] Lone SA, Ahmad A (2020). COVID-19 pandemic—an African perspective. Emerg Microbes Infect.

[3] Carey SA, Afzal A, Jamil A, Williams S, Gottlieb RL (2020). Outpatient COVID-19 surveillance testing in orthotopic heart transplant recipients. Clin Transpl.

[4] Rivinius R, Kaya Z, Schramm R (2020). COVID-19 among heart transplant recipients in Germany: a multicenter survey. Clin Res Cardiol.

[5] Thng ZX, De Smet MD, Lee CS (2020). COVID-19 and immunosuppression: a review of current clinical experiences and implications for ophthalmology patients taking immunosuppressive drugs. Br J Ophthalmol.

[6] Sharma P, Chen V, Fung CM (2020). COVID-19 outcomes among solid organ transplant recipients. Transplantation.

[7] Lee H, Mantell BS, Richmond ME (2020). Varying presentations of COVID-19 in young heart transplant recipients: a case series. Pediatr Transplant.

[8] Duncan MD, Wilkes DS (2005). Transplant-related immunosuppression: a review of immunosuppression and pulmonary infections. Proc Am Thorac Soc.

[9] Li F, Cai J, Dong N (2020). First cases of COVID-19 in heart transplantation from China. J Hear Lung Transpl.

[10] Zhu L, Xu X, Ma K (2020). Successful recovery of COVID-19 pneumonia in a renal transplant recipient with long-term immunosuppression. Am J Transpl.

[11] Qin J, Wang H, Qin X (2020). Perioperative presentation of COVID-19 disease in a liver transplant recipient. Hepatology.

[12] Seminari E, Colaneri M, Sambo M (2020). SARS CoV-2 infection in a renal-transplanted patient: a case report. Am J Transpl.

[13] Liu B, Wang Y, Zhao Y, Shi H, Zeng F, Chen Z (2020). Successful treatment of severe COVID-19 pneumonia in a liver transplant recipient. Am J Transpl.

[14] Chen S, Yin Q, Shi H (2020). A familial cluster, including a kidney transplant recipient, of Coronavirus Disease 2019 (COVID-19) in Wuhan, China. Am J Transpl.

[15] Zhang H, Chen Y, Yuan Q (2020). Identification of kidney transplant recipients with coronavirus disease 2019. Eur Urol.

[16] Wang J, Li X, Cao G, Wu X, Wang Z, Yan T (2020). COVID-19 in a kidney transplant patient. Eur Urol.

[17] Ning L, Liu L, Li W (2020). Novel coronavirus (SARS-CoV-2) infection in a renal transplant recipient: case report. Am J Transpl.

[18] Bussalino E, De Maria A, Russo R, Paoletti E (2020). Immunosuppressive therapy maintenance in a kidney transplant recipient with SARS-CoV-2 pneumonia: a case report. Am J Transpl.

[19] Marx D, Moulin B, Fafi-Kremer S (2020). First case of COVID-19 in a kidney transplant recipient treated with belatacept. Am J Transpl.

[20] Al-Darzi W, Aurora L, Michaels A (2020). Heart transplant recipients with confirmed 2019 novel coronavirus infection: the Detroit experience. Clin Transpl.

[21] García-Cosío MD, Flores Hernán M, Caravaca Pérez P, López-Medrano F, Arribas F, Delgado Jiménez J (2019). Heart transplantation during the coronavirus disease 2019 pandemic: follow-up organization and characteristics of infected patients. Rev Española Cardiol (English Ed 2020).

[22] Fernández-Ruiz M, Andrés A, Loinaz C (2020). COVID-19 in solid organ transplant recipients: a single-center case series from Spain. Am J Transpl.

[23] Pereira MR, Mohan S, Cohen DJ (2020). COVID-19 in solid organ transplant recipients: initial report from the US epicenter. Am J Transpl.

[24] Latif F, Farr MA, Clerkin KJ (2020). Characteristics and outcomes of recipients of heart transplant with coronavirus disease 2019. JAMA Cardiol.

